# Non-neutralizing IgG anti-PID antibodies decreased viral load following high dose vaginal challenge of non-human primates

**DOI:** 10.1186/1742-4690-9-S2-O38

**Published:** 2012-09-13

**Authors:** C Moog, N Dereuddre-Bosquet, M Biedma, S Schmidt, T Decoville, I Mangeot, S Zolla-Pazner, B Vcelar, D Katinger, V Holl, R Le Grand

**Affiliations:** 1 INSERM, Strasbourg, France; 2 CEA, Fontenay-aux-Roses, France; 3 NYU, New York, NY, USA; 4 Polymun Scientific GmbH, Vienna, Austria; 5 Covance, Geneva, Switzerland

## Background

Fc-mediated inhibitory activity of neutralizing antibodies has been shown to participate in HIV protection (Hessell et al. 2007). In addition, a non-neutralizing antibody F240 was found to partially protect macaques from SHIV vaginal transmission (Moore et al., 2011). However, mechanisms involved in this protection need further investigations. In this study, two non-neutralizing antibodies have been selected on the basis of their Fc-mediated inhibitory functions in vitro for further analysis of their protective role on vaginal challenge in non-human primate (NHP).

## Methods

We have assessed and scored various in vitro HIV-inhibitory activities of non-neutralizing antibodies: Fc-mediated inhibition of macrophages through phagocytosis of immunecomplexes, ADCC in primary infected CD4+ T lymphocytes by autologous NK cells, capture of native primary virus particles. Efficacy of two anti-PID antibodies with high in vitro functional scores has been tested in NHP. The combination of two antibodies formulated in 1.6% HEC gel has been topically applied in the vagina of macaques (n=6), 1 hour before vaginal challenge with high dose (10 AID50) of SHIVSF162P3.Infection was followed by assessing viral load in the plasma.

## Results

Although unable to block virus entry at mucosal site as all treated animals became persistently infected, the antibody treated macaques have significant decrease of plasma viral load (day 7, p=0.0479; day 14, p=0.0351, day 42: p=0.0370).

## Conclusion

Decrease in viral load following antibody treatment strongly suggests that non-neutralizing inhibitory antibodies could interfere with early viral replication and dissemination through Fc-mediated inhibitory functions. Additional studies will be required to optimize this inhibition, and combined strategies should be developed to assess the potential synergy between neutralizing and non-neutralizing inhibitory antibodies.

